# Stimulation of neutral lipid synthesis via viral growth factor signaling and ATP citrate lyase during vaccinia virus infection

**DOI:** 10.1128/jvi.01103-24

**Published:** 2024-10-30

**Authors:** Anil Pant, Djamal Brahim Belhaouari, Lara Dsouza, Zhilong Yang

**Affiliations:** 1 Department of Veterinary Pathobiology, College of Veterinary Medicine & Biomedical Sciences, Texas A&M University, College Station, Texas, USA; 2 Division of Biology, Kansas State University, Manhattan, Kansas, USA; Michigan State University, East Lansing, Michigan, USA

**Keywords:** poxvirus, vaccinia virus, metabolism, EGFR, ATP citrate lyase, fatty acids, lipid droplets

## Abstract

Fatty acid metabolism can provide various products essential for viral infections. How vaccinia virus (VACV), the prototype of poxviruses, modulates fatty acid metabolism is not well understood. Here, we show that VACV infection results in increased neutral lipid droplet synthesis, the organelles that play a crucial role in storing and mobilizing fatty acids for energy production via β-oxidation. Citrate is the first tricarboxylic acid (TCA) cycle intermediate that can be transported to the cytosol to be converted to acetyl-CoA for *de novo* fatty acid biosynthesis. We found that VACV infection stimulates the S455 phosphorylation of ATP citrate lyase (ACLY), a pivotal enzyme that links citrate metabolism with lipid metabolism. We demonstrate that the inhibition of neutral lipid droplet synthesis and ACLY severely suppresses VACV replication. Remarkably, we found that virus growth factor (VGF)-induced signaling is essential for the VACV-mediated upregulation of ACLY phosphorylation and neutral lipid droplets. Finally, we report that VGF-induced EGFR-Akt pathway and ACLY phosphorylation are important for VACV stimulation of neutral lipid synthesis. These findings identified a new way of rewiring cell metabolism by a virus and a novel function for VGF in the governance of virus-host interactions through the induction of a key enzyme at the crossroads of the TCA cycle and fatty acid metabolism. Our study also provides a mechanism for the role played by VGF and its downstream signaling cascades in the modulation of lipid metabolism in VACV-infected cells.

**IMPORTANCE:**

Neutral lipid droplets are vital players in cellular metabolism. Here, we showed that VACV induces neutral lipid droplet synthesis in infected primary human foreskin fibroblasts and identified the cellular and viral factors needed. We identified VACV encoded growth factor (VGF) as an essential viral factor that induces cellular EGFR-Akt signaling to increase lipid droplets. Interestingly, VACV increases the S455 phosphorylation of ACLY, a key metabolic enzyme that sits at the crossroads of carbohydrate and lipid metabolism in a VGF-EGFR-Akt-dependent manner. We also found that ACLY is vital for VACV-induced lipid droplet synthesis. Our findings identified the modulation of ACLY by a virus and identified it as a potential target for antiviral development against pathogenic poxviruses. Our study also expands the role of growth factor signaling in boosting VACV replication by targeting fatty acid metabolism.

## INTRODUCTION

Poxviruses have significant impacts on public health because of their ability to cause illness and death in both humans and animals. The current global outbreak of mpox, which has been reported in 118 countries with more than 97,000 cases (including over 32,000 in the USA as of June 12, 2024), highlights the potential for poxviruses to cause a pandemic (1). In addition, despite the successful eradication of smallpox, one of the most devastating diseases in human history, there is still a risk of its re-emergence, posing a serious threat to national security (2, 3). On the other hand, many poxviruses are utilized as vaccine carriers and oncolytic virotherapy agents to combat other diseases (4–6). Vaccinia virus (VACV) serves as the prototype poxvirus and is highly relevant for studying highly pathogenic poxviruses such as mpox and smallpox viruses, given their high genomic similarity with over 95% identical sequences (7).

During viral infections, metabolism becomes a battleground between host cells and viruses. Viruses alter the metabolic landscape of infected cells, utilizing host cell resources for replication (8, 9). Despite significant interest, the mechanisms behind this viral hijacking of host cell metabolism remain largely unknown. Understanding virus-induced metabolic regulation offers the potential for novel antiviral strategies and insights into fundamental cellular processes. VACV induces profound alterations in host cell metabolism including the tricarboxylic acid (TCA) cycle and fatty acids. We have previously shown that VACV infection elevates the levels of citrate and other intermediates of the TCA cycle and modulates metabolites closely related to the TCA cycle (10). VACV selectively upregulates the translation efficiency of oxidative phosphorylation (OXPHOS) mRNAs, indicating the requirement for an increased and continuous supply of energy during virus replication (11). Moreover, VACV depends on *de novo* fatty acid synthesis to generate an energy-favorable environment (12), suggesting the virus may need to modulate fatty acid synthesis. Interestingly, however, our global metabolic profiling of the VACV-infected primary human foreskin fibroblast (HFF) cells showed a decrease in the levels of acetyl-CoA and long-chain fatty acids and an increase in the levels of carnitine-conjugated fatty acids that are essential for the generation of energy via β-oxidation (10).

**Fig 1:**
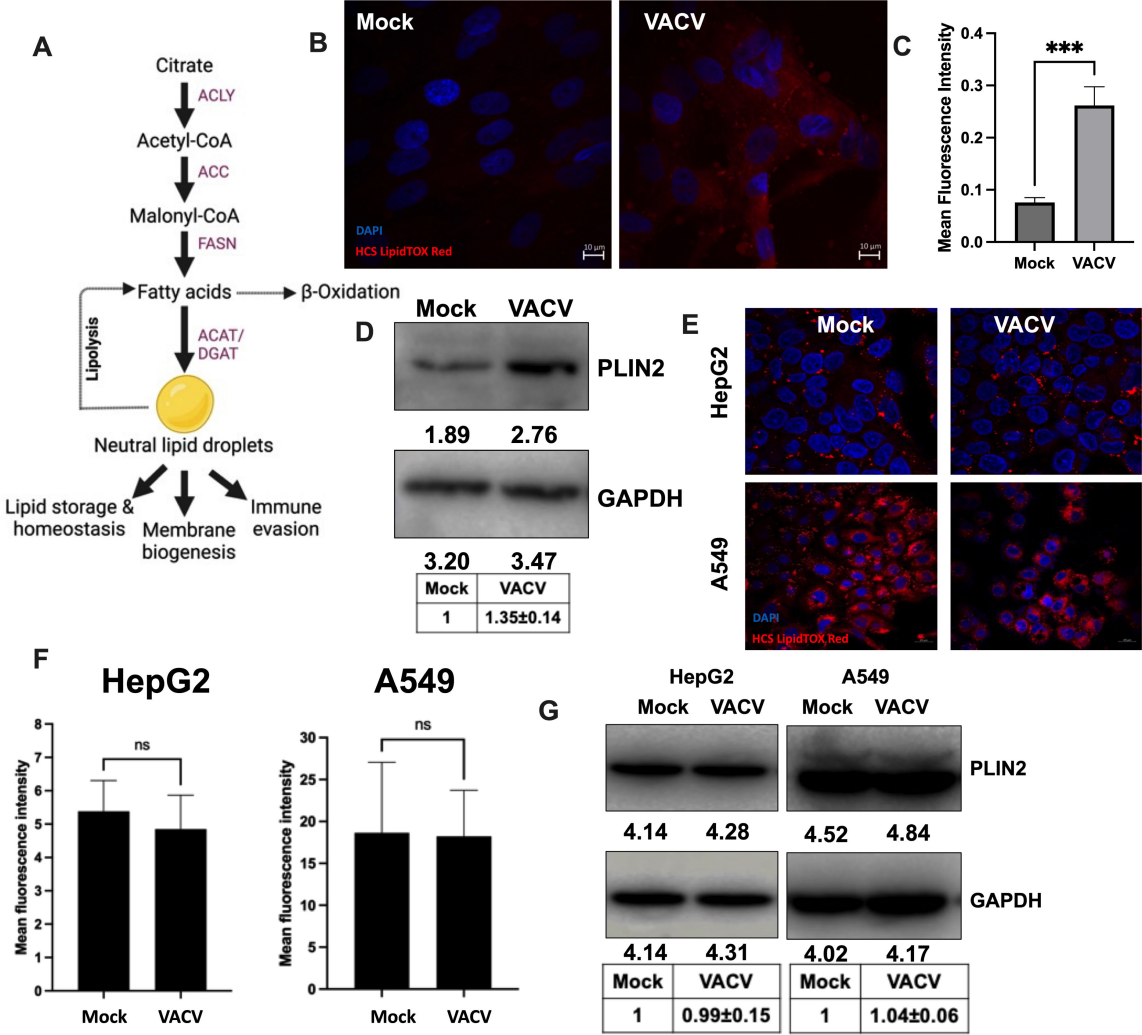
VACV stimulates neutral lipid droplet formation in primary HFFs. (**A**) Role of neutral lipids in cellular metabolism. The enzyme ACLY converts cytoplasmic citrate, generated by the TCA cycle from glucose or glutamine, into acetyl-CoA and oxaloacetate. Acetyl-CoA can be further utilized for lipid synthesis, and neutral lipid synthesis via a series of reactions catalyzed by the enzymes ACC, FASN, ACAT, and DGAT. The fig was created with BioRender.com. (**B**) VACV infection stimulates the formation of neutral lipid droplets. HFFs were infected with VACV at an MOI of 5 for 8 h. Lipid droplets were stained with HCS Lipidtox Red, and the nuclei were stained with DAPI and imaged under a confocal microscope (*n* ≥ 3). (**C**) The intensities of the red signals corresponding to the lipid droplets in Fig. 1B were quantified in the bar graph using ImageJ. (**D**) VACV infection increases the lipid droplet-associated protein PLIN2 levels in HFFs. HFFs were infected with WT VACV at an MOI of 5 for 8 h. Western blotting analysis was performed to measure the levels of PLIN2. (**E**) VACV infection-induced lipid droplet formation is cell-type specific. HepG2 and A549 cells were infected with VACV at an MOI of 5 for 8 h. Lipid droplets were stained with HCS Lipidtox Red, and the nuclei were stained with DAPI and imaged under a confocal microscope (*n* ≥ 3). (**F**) The intensities of the signals corresponding to the lipid droplets in Fig. 1E were quantified in the bar graph. (**G**) VACV infection does not alter the levels of PLIN2 in HepG2 and A549 cells. The cells were infected with WT VACV at an MOI of 5 for 8 h. Western blotting analysis was performed to measure the levels of PLIN2. For *P* values, ns, *P* >  0.05; **P  ≤ 0.01; ***P  ≤  0.001. ****P ≤ 0.0001. In D and G, representative images of multiple biological replicates were shown. The numbers below each band indicate the average intensities of respective proteins as calculated by ImageJ. The relative average intensities ± standard deviation of PLIN2/GAPDH, normalized to mock, are shown in the tables below the images (*n* ≥ 3 for D, *n* ≥ 2 for G). n indicates the number of biological replicates.

Neutral lipid droplets serve as energy reserves and contribute to maintaining lipid homeostasis by connecting fatty acid metabolism and β-oxidation, thereby ensuring proper cellular function and survival (13). Neutral lipid metabolism is important during virus replication for various purposes including maintaining lipid homeostasis, membrane biogenesis, and immune evasion (14–16) (Fig. 1A). In addition to relying on lipid metabolism for energy (12), VACV relies on the cholesterol content of lipid rafts during membrane fusion for entry and egress (17). Moreover, VACV proteins often undergo fatty acylation modifications, such as palmitoylation and myristoylation, which are essential for their proper function (18, 19). Since VACV is an enveloped virus, it relies on host cell lipids for the generation and composition of the viral membrane. A shotgun lipidomic analysis of VACV membranes showed that neutral lipids made up about 20% of the total lipids measured (20), suggesting a potentially essential role of neutral lipids in viral envelop formation. Because all of the aspects of lipid metabolism can be linked to neutral lipid droplets, it would only be logical to think that VACV modulates the neutral lipid metabolism upon infection. In fact, a recent paper showed increased lipid droplet formation during VACV infection of mouse bone marrow-derived macrophages (21).

Citrate, which is the first metabolite produced by the TCA cycle, can be shuttled out of the mitochondria to generate acetyl-CoA (Fig. 1A) (22, 23). Acetyl-CoA represents a key precursor of fatty acid biosynthesis and serves as an important source of the acetyl groups for histone acetylation (24). The conversion rate from citrate to acetyl-CoA is governed by the enzyme ATP citrate lyase (ACLY) (23). Therefore, ACLY links carbohydrate metabolism (glycolysis and the TCA cycle), glutamine metabolism (reductive carboxylation), fatty acid synthesis, and histone acetylation, making it a pivotal enzyme in cellular metabolism (25, 26). ACLY can be phosphorylated at least three different sites: threonine 446 (T446), serine 450 (S450), and serine 455 (S455, in humans and mice) (27). Phosphorylation of ACLY increases enzymatic activity (28). The expression and activity of ACLY are significantly upregulated in several malignancies such as bladder, breast, lung, liver, stomach, prostate, and colon cancers (25, 29–33), and the overexpression of ACLY correlates with poor prognosis in lung adenocarcinoma and blood cancers (30, 34). In addition, the chemical and genetic suppression of ACLY has been shown to inhibit the proliferation and progression of various cancers (35). Because ACLY acts at a critical juncture of host metabolism, ACLY expression levels could be affected by many viruses. However, the mechanisms through which a viral infection may modulate this key host metabolic enzyme and its consequences are lacking.

Here, we report that VACV infection stimulates the synthesis of lipid droplets in human foreskin fibroblasts (HFFs). Lipid droplets are rich in neutral lipids and play an important role in the storage of lipids as the source to fuel the TCA cycle for energy production. We found that neutral lipid droplet synthesis is important for VACV replication. We also report that VACV infection induces the S455 phosphorylation of ACLY, and chemical and genetic inhibition of ACLY severely suppresses VACV replication. VGF-induced growth factor signaling and the Akt pathway are essential for the VACV-mediated upregulation of ACLY phosphorylation. Finally, we report that VGF-induced ACLY phosphorylation via the EGFR-Akt pathway is important for the synthesis of lipid droplets during VACV infection. These findings identified a novel function for VGF in the governance of virus-host interactions through the induction of a key enzyme associated with host fatty acid metabolism. Our study also provides the mechanism by which VGF and its downstream signaling cascades modulate lipid metabolism during VACV infection. Furthermore, our findings lay the foundation and expand the understanding of the role played by growth factors in the regulation of cellular metabolism during viral infections.

## MATERIALS AND METHODS

### Cells and viruses

Primary HFFs were a kind gift from Dr. Nicholas Wallace at Kansas State University. Primary HFFs, HeLa cells (ATCC CCL-2), and A549 (ATCC CCL-185) were grown in Dulbecco’s modified Eagle medium (DMEM; Fisher Scientific), supplemented with 10% fetal bovine serum (FBS; Peak Serum), 2 mM glutamine (VWR), 100 U/mL of penicillin, and 100 µg/mL streptomycin (VWR) in a humidified incubator at 37°C with 5% CO_2_. BS-C-1 cells (ATCC CCL-26) and HepG2 cells (kindly provided by Dr. Annie Newell-Fugate at Texas A&M University) were cultured in Eagle’s minimal essential medium (EMEM; Fisher Scientific) using the same supplements and environments described for HFF culture. The WR strain of VACV (ATCC VR-1354) was amplified, purified, and quantified using previously described titration methods (36). When the cells reached the confluency of approximately 90%–95%, they were infected with the desired MOI in DMEM (Fisher Scientific) lacking glucose, L-glutamine, L-asparagine, sodium pyruvate, and phenol red, which was supplemented with 2% dialyzed FBS (Gibco), 100 U/mL of penicillin, and 100 µg/mL streptomycin (VWR). The medium was further supplemented with 1 g/L glucose (Fisher Scientific), glucose plus 2 mM glutamine, or acetate as required.

The vΔVGF and vΔVGF_Rev mutant VACVs were generated as previously described (10). Briefly, VGF-deleted VACV was created through homologous recombination by replacing the VGF-encoding C11R gene with a green fluorescent protein (GFP) gene. The GFP coding sequence, driven by a P11 promoter and flanked by 500 bp homologous sequences upstream and downstream of the C11R gene, was constructed using overlapping PCR and transfected into VACV-infected HeLa cells. Recombinant viruses expressing GFP were harvested from HeLa cells and plaque purified in BS-C-1 cells. The recombinant vΔVGF, with the deletion of two copies of the C11R gene at both ends of the virus genome, was confirmed by PCR. The C11R revertant recombinant VACV vΔVGF_Rev was generated similarly by inserting a DNA fragment containing one copy of the C11R gene under the C11 promoter, followed by the dsRED coding sequence under a P11 promoter, between the VACWR146 and VACWR147 loci in the central region of the VACV genome.

### Antibodies and chemicals

Antibodies against VACV proteins E3, D13 (37), and A17 (38) were a kind gift from Dr. Yan Xiang and Dr. Bernard Moss. Antibodies against phospho-ACLY (S455), total ACLY, phospho-Akt (S473), total Akt, perilipin 2, phospho-ACC (S79), total ACC, β-tubulin, ACAT (SOAT1), and horseradish peroxidase-conjugated secondary antibodies were purchased from Cell Signaling Technology. Antibody against the mitochondrial citrate transporter SLC25A1 was purchased from Proteintech. The anti-glyceraldehyde-3-phosphate dehydrogenase (anti-GAPDH) and DGAT1 antibodies were purchased from Santa Cruz Biotechnology.

The ACLY inhibitor SB 204990, BMS-303141, NDI-091143, and the DGAT inhibitor PF-06424439 were purchased from Cayman Chemicals. Sodium acetate powder for cell culture, cytosine-1-β-d-arabinofuranoside (AraC), and cycloheximide (CHX) were purchased from Sigma-Aldrich. Other chemical inhibitors, including MK-2206 2HCl, blebbistatin, afatinib, avasimibe, and T-863 were purchased from Selleck Chemicals and used at the indicated concentrations.

### Cell viability assays

Cell viability assays were performed using a hemocytometer and the trypan blue exclusion assay, as described previously (39). Briefly, after performing each indicated treatment for the indicated time, cells grown in a 12-well plate were harvested with 300 µL trypsin and mixed with 500 µL DMEM using a micropipette. Equal volumes (20 µL) of the cell suspension and 4% trypan blue (VWR) were gently mixed, and the numbers of live and dead cells in each condition were counted using a hemocytometer or an automated cell counter.

### Western blotting analysis

Western blotting was performed as previously described (40). Briefly, after the indicated treatment for the indicated time, the cells were lysed in NP-40 cell lysis buffer and reduced with 100 mM dithiothreitol (DTT), followed by denaturation in sodium dodecyl sulfate-polyacrylamide gel electrophoresis (SDS-PAGE) loading buffer. The samples were boiled at 99°C for 5 min and separated by SDS-PAGE, followed by transfer to a polyvinylidene difluoride (PVDF) membrane. Membrane blocking was performed for 1 h at room temperature in 5% bovine serum albumin (BSA; VWR) in Tris-buffered saline containing Tween-20 (TBST). The indicated primary antibodies were diluted in the BSA-blocking buffer and incubated overnight at 4°C. After three washes with TBST for 10 min, the membrane was incubated with horseradish peroxidase-conjugated secondary antibody for 1 h at room temperature. Finally, the membranes were developed with Thermo Scientific SuperSignal West Femto Maximum Sensitivity Substrate and imaged using a c300 Chemiluminescent Western Blot Imaging System (Azure Biosystems). If western blotting analysis using another antibody was required, the antibodies were stripped from the membrane by Restore (Thermo Fisher Scientific, Waltham, MA, United States), and the processes of blocking, incubation with another primary antibody, secondary antibody, and imaging were repeated.

The quantification of the intensities of the bands in Western blot images was performed using the software ImageJ2 (Fiji; version 2.9.0/1.53t) (41). First, the individual figure files were imported in tiff format and the background was subtracted to minimize the noise. Thereafter, the file was converted to a grayscale 8-bit image and the image was inverted. The rectangular selection tool was then used to select the first band as the first region of interest (ROI) and its intensity was measured. Accordingly, the rectangular selection tool was moved to the next ROI and the measurement process was repeated until all the bands were quantified. The average intensity of the individual bands from biological repeats is reported under respective lanes. Furthermore, for normalization purposes, first, the intensity of each respective band of indicated protein was divided by the intensity of the loading control of the same treatment. Then, this output value was further normalized to mock or vehicle conditions (as indicated in individual figures), by dividing each value by the value of the mock or vehicle condition.

### VGF and EGF peptide treatment

The EGF peptide was purchased from Cell Signaling Technologies and used at indicated concentrations. The VGF peptide was also expressed and purified by GenScript using a HD CHO-S cell mammalian expression system. The C-terminal 6xHis tagged peptide sequence of the cleaved, secreted portion of the VGF peptide (42) was generated and used at indicated concentrations. The VGF peptide sequence with 6xHis tag used (43) is as follows: DSGNAIETTSPEITNATTDIPAIRLCGPEGDGYCLHGDCIHARDIDGMYCRCSHGYTGIRCQHVVLVDYQRSENPNTHHHHHH.

### RNA interference

Specific siRNAs for the indicated target genes and the negative control siRNAs were purchased from Qiagen. The siRNAs were mixed in Lipofectamine RNAiMAX transfection reagent (Fisher Scientific) and transfected to the HFFs in a six-well plate at a final concentration of 5 nm in OPTIMEM media as per the manufacturer’s instructions. After 48 h, the efficiency of knockdown was confirmed using a western blotting assay.

### VACV entry assay

A quantitative real-time PCR (qPCR)-based assay by quantifying viral DNA was used as to compare the amount of virus entering the cells. Briefly, a day before the assay, HFFs were plated on a six-well plate. On the day of the assay, the cells were infected with an MOI of 2 of VACV in the presence or absence of indicated compounds. The cells were incubated at 37°C for 1 h to allow for the viral entry. Then the cells were washed 3 times with 1× phosphate-buffered saline (PBS) to remove any unattached virus and trypsinized to extract DNA using the E.Z.N.A SQ Blood DNA Kit from Omega Bio-tek. The following primers were used to detect the relative levels of VACV DNA in the cells by qPCR using a CFX96 real-time PCR instrument (Bio-Rad, Hercules, CA) and the All-in-one 2× qPCR mix (GeneCopoeia).

C11 forward primer: AAACACACACTGAGAAACAGCATAAA

C11 reverse primer: ACTATCGGCGAATGATCTGATTATC

18S rRNA forward primer: CGATGCTCTTAGCTGAGTGT

18S rRNA reverse primer: GGTCCAAGAATTTCACCTCT

The following settings were used for the qPCR assay: Initial denaturation at 95°C for 10 min, followed by 39 cycles of denaturation at 95°C for 10 s, annealing and reading fluorescence at 53°C for 30 s, and extension at 72°C for 15  s. 18sRNA was used as an internal control for normalization.

### *Gaussia* luciferase assay

The *Gaussia* luciferase activities in the cell culture supernatant were measured as previously described (44). In short, cells were infected with recombinant VACV encoding *Gaussia* luciferase under the control of viral early (C11R; vEGluc), intermediate (G8R; vIGluc), or late (F17R; vLGluc) promoters. At the indicated time points, the cell culture media was collected, and luciferase activities were measured using a Pierce *Gaussia* luciferase flash assay kit (Thermo Scientific) and a luminometer.

### VACV plaque assay

The monolayers of BS-C-1 cells were infected with VACV. One-h post-infection, the media was changed to EMEM-containing supplements as described above plus 0.5% methylcellulose (Fisher Scientific). The viruses were allowed to grow and form plaque for 48 h after which the growth medium was discarded, and the cells were treated with 0.1% (wt/vol) crystal violet (Fisher Scientific) in 20% ethanol for 10 min. The number of plaques was counted to calculate the titer of VACV.

### Detection of lipid droplets

The cells were seeded on the ibidi 12-well Chamber at a density of 10,000 cells per well. After the cells reached confluency, they were washed with PBS and infected with the indicated MOI of WT-VACV or vΔVGF in special DMEM media (Fisher Scientific) with 2% dialyzed FBS, 2 mM glutamine, and 1 g/L glucose. After incubation for appropriate time in a humidified incubator at 37°C and 5% CO_2_ the cells were fixed with 4% paraformaldehyde for 10 min at RT. The fixative solution was removed, and the cells were gently washed with 1× PBS buffer 2–3 times to remove residual formaldehyde. The 1,000× LipidTOX red neutral lipid stain was diluted 1:1,000 in 1× PBS buffer to make a 1× solution and 100 μL of the diluted stain was added to each well. The cells were then incubated for 1 h at RT and then washed thrice with 1× PBS before being incubated with 1 µg/mL 2-(4-amidinophenyl)-1H-indole-6-carboxamidine (DAPI) for 20  min at RT in the dark. Finally, the cells were washed three more times with 1× PBS on the coverslips, the silicone gasket was removed, and the culture chamber was mounted on a glass coverslip using ProLong gold mounting medium. The slides were stored at 4°C until confocal microscopy analyses were performed using Zeiss LSM 780 confocal microscope. Processing of microscopy images was performed with Zeiss Zen 3.1 (blue edition) and the images were quantified using ImageJ2.

The mean fluorescence intensity was determined by analyzing multiple randomly captured images, all obtained using the same confocal parameters. All the experiments were performed at least three times and at least five different random fields were captured per treatment. We utilized ImageJ2 software for this analysis. First, each image was loaded into ImageJ2 and the software’s measurement tool was used to estimate the mean fluorescence intensity. To ensure statistical accuracy, the MFI analysis was conducted with approximately the same number of cells for each condition. The count was done with at least 70 cells per condition across at least five fields. This approach was consistently applied to all confocal figures to maintain statistical rigor. For quantification, we employed the integrated measurement tools available within ImageJ2, which provide the average fluorescence intensity across the selected ROIs. The mean fluorescence intensity for each condition was then used to perform statistical comparisons through ANOVA testing.

### Statistical analyses

Unless otherwise stated, the data presented represent the mean of at least three biological replicates. Data analysis was performed in GraphPad Prism (version 10.2.3 (347)) and Microsoft Excel (version 16.43). A two-tailed paired *t-*test was used to evaluate any significant differences between the two means. For the comparison of the means of more than two groups, one-way ANOVA was performed followed by Tukey’s Honest Significant Difference (HSD) or Dunnett’s test. For the comparison of the difference between the two factors [infection status (infected vs. uninfected) and treatment type (vehicle vs. compound)] a two-way ANOVA was performed followed by a Tukey’s HSD. The error bars indicate the standard deviation of the experimental replicates. The following convention for symbols was used to indicate statistical significance: ns (not significant), *P* >  0.05; *P  ≤ 0.05; **P ≤ 0.01; ***P ≤ 0.001; ****P ≤ 0.0001.

## RESULTS

### VACV infection induces lipid droplet formation in HFFs, which is required for efficient VACV replication

Our previous study using global metabolic profiling of VACV-infected HFFs revealed a decrease in the long-chain fatty acids but an increase in carnitine-conjugated fatty acids, which are important for energy generation via β-oxidation (10). Lipid droplets, the cellular organelles that store neutral lipids generated from fatty acids (Fig. 1A), are a major source of energy production through β-oxidation (13, 14). We measured the neutral lipids in the VACV-infected HFFs using HCS LipidTOX neutral lipid staining. The wild-type VACV(WT-VACV) infection substantially increased host cell neutral lipid droplets (Fig. 1B and C). A recent study also reported that VACV infection increases lipid droplets in mouse bone marrow-derived macrophages (21). Perilipin 2 (PLIN2) is a protein that coats intracellular lipid storage droplets (45, 46). Although traditionally thought to be expressed only in adipocytes, emerging studies have identified an important role of this protein in non-adipocytic cells such as fibroblasts (47). We found that VACV infection increased the levels of PLIN2 in HFFs (Fig. 1D).

We further examined the effect of VACV infection on lipid droplet formation in human hepatoma cell lines (HepG2) and human lung cancer cell lines (A549). Unlike in the primary HFFs, VACV infection of HepG2 cells or A549 cells did not increase the HCS LIPIDTOX Red staining (Fig. 1E and F). Moreover, we found that VACV infection did not increase the levels of PLIN2 in either of these two transformed cell lines (Fig. 1G). These results indicate that the lipid droplet synthesis during VACV infection could be cell-type dependent. The transformed cells like HepG2 and A549 may already have a hyperactive lipid metabolism and increased basal lipid droplets compared to the primary cells such as the HFFs and may respond differently to VACV infection.

### Neutral lipid droplet synthesis is important for VACV replication

**Fig 2:**
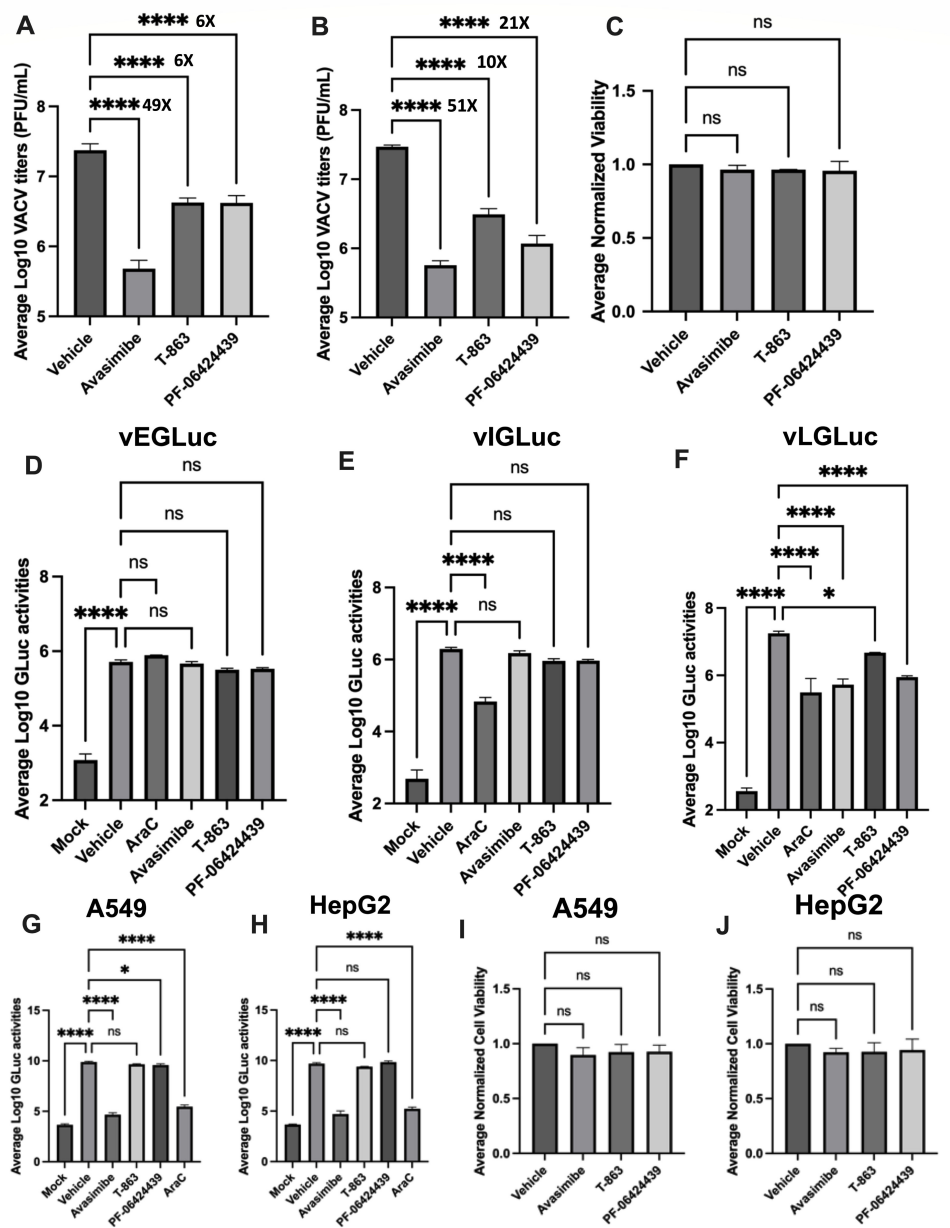
Neutral lipid synthesis is important for efficient VACV replication. (**A, B**) Chemical inhibition of lipid droplet synthesis suppresses VACV replication. HFFs were infected with VACV in the presence or absence of 30 µM avasimibe, 125 µM T-863, or 125 µM PF-06424439. Virus titers were measured by a plaque assay at (**A**) 24 hpi (MOI = 2) and (**B**) 48 hpi (MOI = 0.01). The numbers above the bars indicate the fold reduction in VACV titers compared to vehicle (*n* ≥ 3). (**C**) The inhibition of lipid droplet synthesis does not alter HFF viability. HFFs were grown in the presence or absence of 30 µM avasimibe, 125 µM T-863, or 125 µM PF-06424439 for 48 h. Cell viability was determined by trypan blue exclusion assay using a hemocytometer (*n* ≥ 3). (**D-F**) Lipid droplet synthesis is important for the expression of VACV late genes. HFFs were infected with VACV (MOI = 2) that expressed *Gaussia* luciferase under (**D**) early (vEGLuc), (**E**) intermediate (vIGLuc), and (**F**) late (vLGLuc) promoters. A *Gaussia* luciferase assay was performed to measure the early, intermediate, and late gene expression at 6, 8, and 16 hpi, respectively. AraC, which inhibits VACV DNA replication and intermediate and late gene expression, was used at a concentration of 40 µg/mL as a control (*n* ≥ 3). (**G, H**) Chemical inhibition of lipid droplet synthesis differentially suppresses VACV replication in transformed cells. HepG2 (**G**) and A549 (**H**) cells were infected with MOI of 0.01 of vLGLuc in the presence or absence of 30 µM avasimibe, 125 µM T-863, or 125 µM PF-06424439. A *Gaussia* luciferase assay was performed to measure the late gene expression at 24 hpi. AraC was used at a concentration of 40 µg/mL as a control (*n* ≥ 3). (**I, J**) the inhibition of lipid droplet synthesis does not alter the viability of the transformed cells. HepG2 (**I**) and A549 (**J**) cells were grown in the presence or absence of 30 µM avasimibe, 125 µM T-863, or 125 µM PF-06424439 for 48 h. Cell viability was determined by trypan blue exclusion assay using an automated cell counter (*n* ≥ 3). n indicates the number of biological replicates. For *P*-values, ns, *P*  >  0.05; *P  ≤  0.05; ****P ≤ 0.0001.

Neutral lipid droplets are synthesized in a multi-step process that is regulated at different steps by different enzymes (Fig. 1A). In the cytoplasm, citrate is converted to acetyl-CoA by ACLY (23). Acetyl-CoA carboxylase (ACC) then catalyzes the carboxylation of acetyl-CoA to malonyl-CoA (48), which serves as a substrate for fatty acid synthesis mediated by fatty acid synthase (FASN) (49). These synthesized fatty acids are subsequently esterified to CoA by acyl-CoA synthetase, then converted to triglycerides by acyl-CoA: diacylglycerol acyltransferase (DGAT), ultimately forming neutral lipid droplets (50). In addition, acetyl-CoA can also be converted to cholesterol esters by acyl-CoA: cholesterol acyltransferase (ACAT), contributing to the formation of lipid droplets (51). To examine the role of lipid droplet formation in VACV replication, we treated HFFs with specific inhibitors of ACAT [avasimibe (52)] and DGAT [T863, for DGAT1 (53), PF-06424439 for DGAT2 (54)], and assessed VACV replication. We found that the chemical inhibitors of the ACAT and DGAT significantly reduced VACV titers (Fig. 2A and B) without affecting the cell viability (Fig. 2C).

VACV genes are expressed sequentially in a cascade manner. Following entry, early genes of VACV are immediately transcribed, leading to DNA replication, followed by sequential expression of intermediate genes and late genes (7). To determine the stage of viral replication affected, HFFs were infected with one of three reporter VACVs containing a secreted *Gaussia* luciferase gene under viral early (vEGluc), intermediate (vIGluc), and late (vLGluc) promoters in the presence or absence of the inhibitors of ACAT and DGAT. *Gaussia* luciferase activities in the cell culture medium were then measured to assess viral gene expression. We found that these inhibitors suppress the expression of late genes without significantly affecting the intermediate or the early gene expression (Fig. 2D through F). We further tested the effects of inhibition of ACAT and DGAT in VACV replication in HepG2 and A549 cells using the *Gaussia* luciferase reporter assay. The inhibition of ACAT (by avasimibe) significantly reduced VACV replication in both the cell lines (Fig. 2G and H). Interestingly, however, the inhibitors of DGAT (T863 and PF-06424439) did not affect the replication of VACV in either of the cells (Fig. 2G and H). None of the compounds affected the viability of the HepG2 or A549 cells at the concentration used in these experiments (Fig. 2I and J). This discrepancy in the decrease in VACV replication upon inhibition of the ACAT and DGAT in different cell lines further highlights our assumption that modulation of the lipid droplet synthesis during VACV infection could be cell-type dependent and that different cell types could react differently to the virus infection. Nevertheless, these findings highlight the importance of the synthesis of neutral lipid droplets in VACV replication in primary HFFs.

### VACV infection stimulates ACLY S455 phosphorylation

**Fig 3:**
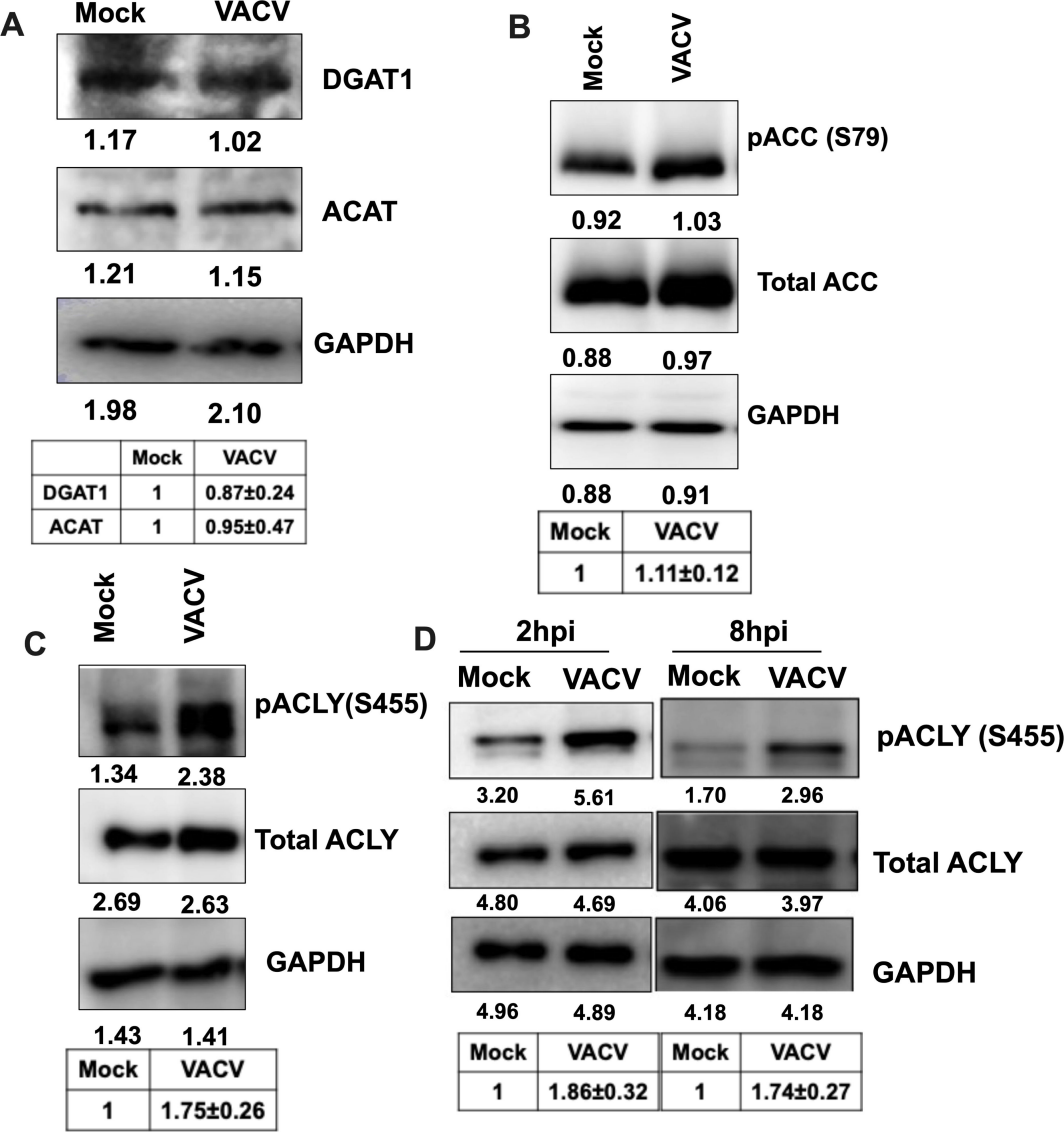
VACV infection induces ACLY S455 phosphorylation in HFFs. (**A**) The protein levels of ACAT and DGAT are unaffected in HFFs upon VACV infection. HFFs were infected with VACV at an MOI of 5. Western blotting analysis was performed to measure the levels of ACAT and DGAT1 at 4 hpi. (**B**) VACV infection does not enhance the phosphorylation of ACC. HFFs were infected with MOI-5 of WT VACV. Western blotting analysis was performed to measure the levels of indicated proteins at 4 hpi. (**C**) VACV infection induces the activation of ACLY phosphorylation at serine 455. HFFs were infected with VACV at an MOI of 5. Western blotting analysis was performed to measure the levels of ACLY at 4 h post-infection (hpi). (**D**) The upregulation of ACLY S455 phosphorylation can be observed early during VACV infection. HFFs infected with WT VACV at an MOI of 5. The samples were collected at 2 hpi and 8 hpi, followed by Western blotting analysis. Representative images of multiple biological replicates were shown. The numbers below each band indicate the average intensities of respective proteins as calculated by ImageJ. The relative average intensities ± standard deviation of ACAT/GAPDH or DGAT1/GAPDH (A, *n* ≥ 3), pACC/GAPDH (B, *n* ≥ 2), pACLY/GAPDH, normalized to mock, are shown in the tables below the images (C and D, *n* ≥ 3). n indicates the number of biological replicates.

Next, we examined the effect of VACV infection on the levels of the key enzymes of the neutral lipid droplet synthesis pathway listed in Fig. 1A. Western blotting analysis showed no noticeable difference in the protein levels of the two enzymes ACAT and DGAT upon VACV infection (Fig. 3A). A previous study by Greseth et al. reported that VACV infection did not result in increased FASN levels (12). The study, however, showed that inhibition of FASN significantly reduced VACV replication, highlighting the importance of this step in VACV replication. We also did not observe a substantial difference in the phosphorylated (inactive) or the total levels of ACC (55) (Fig. 3B).

We previously showed that VACV infection increases the levels of citrate (10) and other TCA cycle intermediates in primary human foreskin fibroblasts (HFFs). Citrate can be transported out of the mitochondria into the cytosol, where it is converted to acetyl-CoA and oxaloacetate (OAA), and acetyl-CoA serves as a precursor for fatty acid biosynthesis (26). The conversion of citrate to acetyl-CoA is catalyzed by the enzyme ACLY (Fig. 1A) (23). It has been suggested that post-translational phosphorylation, including the phosphorylation at S455, is one of the post-translational mechanisms to increase the enzymatic activity of ACLY (28, 56, 57). Remarkably, we found that VACV infection increased ACLY phosphorylation at S455 in HFFs (Fig. 3C). Moreover, we found that VACV infection increased ACLY phosphorylation was observable at 2- and 8 h post-infection (hpi) (Fig. 3D), indicating that VACV can modulate ACLY activity starting early during the infection.

### Inhibition of ACLY suppresses VACV replication

**Fig 4:**
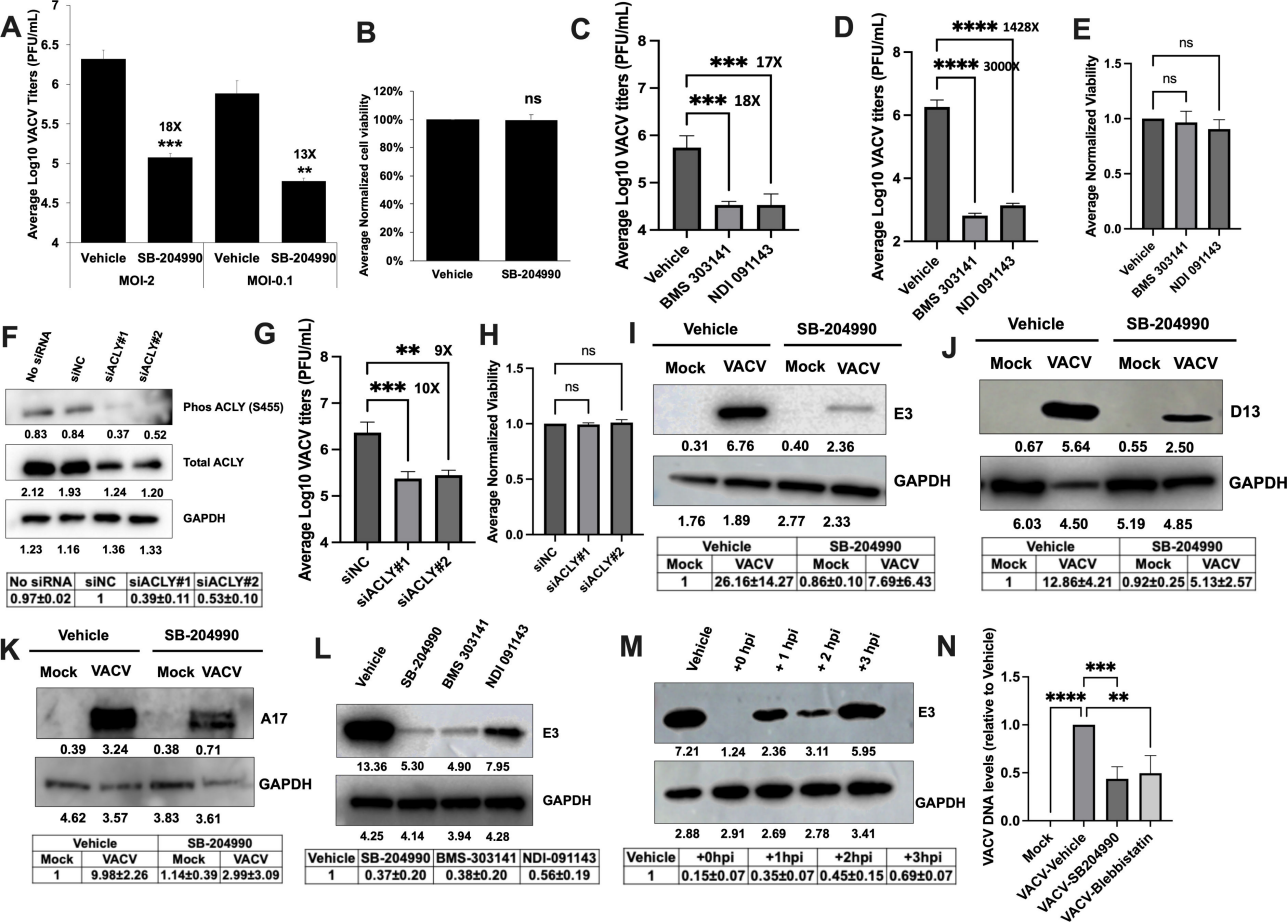
Inhibition of ACLY suppresses VACV replication. (**A**) Chemical inhibition of ACLY suppresses VACV replication. HFFs were infected with VACV in the presence or absence of 100 µM SB-204990. Virus titers were measured by plaque assay at 24 hpi (MOI = 2) and 48 hpi (MOI = 0.1). The numbers above the bars indicate the fold reduction in titers compared to vehicle (*n* ≥ 3). (**B**) The inhibition of the ACLY does not alter HFF viability. HFFs were grown in the presence or absence of 100 µM SB-204990 for 48 h. Cell viability was determined by trypan blue exclusion assay using a hemocytometer (*n* ≥ 3). (**C, D**) Other chemical inhibitors of ACLY effectively inhibit VACV replication. HFFs were infected with WT VACV in the presence or absence of 10 µM BMS-303141 or 20 µM NDI-091143. Virus titers were measured by a plaque assay at (**C**) 24 hpi (MOI = 2) or (**D**) 48 hpi (MOI = 0.01). The numbers above the bars indicate the fold reduction in titers compared to vehicle (*n* ≥ 3). (**E**) The ACLY inhibitors do not alter HFF viability. HFFs were cultured in the presence or absence of 10 µM BMS-303141 or 20 µM NDI-091143 for 48 h. Cell viability was determined by trypan blue exclusion assay (*n* ≥ 3). (**F**) siRNA-mediated knockdown of ACLY. HFFs were transfected with a negative control siRNA or two specific siRNAs targeting ACLY for 48 h. Western blotting was performed to measure the levels of ACLY protein expression. (**G**) siRNA-mediated knockdown of ACLY decreases VACV infection. HFFs were transfected with the indicated siRNAs for 48 h and infected with WT VACV at an MOI of 2. Viral titers were measured at 24 hpi. The numbers above the bars indicate the fold reduction in titers compared to siNC control (*n* ≥ 3). (**H**) ACLY knockdown does not affect HFF viability. HFFs were transfected with the indicated siRNAs for 48 h. Cell viability was determined by trypan blue exclusion assay (*n* ≥ 3). (**I–K**) Chemical inhibition of ACLY suppresses VACV protein synthesis. HFFs were infected with the VACV at an MOI of 5 in the absence or presence of 100 µM SB-204990. Western blotting analysis was performed to measure the levels of (**I**) early protein (**E3**) at 4 hpi, (**J**) intermediate protein (**D13**) at 8 hpi, and (**K**) late protein (**A17**) at 16 hpi. (**L**) Other chemical inhibitors of ACLY also suppress VACV early protein levels. HFFs were infected with VACV at an MOI of 5 in the absence or presence of 100 µM SB-204990, 10 µM BMS-303141, or 20 µM NDI-091143 for 4 h. Western blotting analysis was performed to measure the levels of early protein (**E3**). (**M**) ACLY inhibition possibly affects VACV at or prior to VACV early gene expression. HFFs were infected with the VACV at an MOI of 5. ACLY inhibitor SB-204990 was added at 100 µM concentration at 0 (the time of infection), 1, 2, or 3 hpi. Samples were collected at 4 hpi and a Western blotting analysis was performed to measure the levels of VACV early protein (**E3**). (**N**) ACLY inhibition reduces VACV entry into the cells. HFFs were infected with VACV at an MOI of 2 in the presence of 100 µM SB-204990 or vehicle. The VACV entry inhibitor blebbistatin was used as a positive control at a concentration of 75 µM. At 1 hpi, the cells were washed, trypsinized and lysed to measure viral DNA levels (*n* ≥ 3). Error bars represent the standard deviation of at least three biological replicates. For *P* values, ns, *P* >  0.05; **P ≤ 0.01; ***P ≤ 0.001; ****P ≤  0.0001. For Western blot, representative images of multiple biological replicates were shown. The numbers below each band indicate the average intensities of respective proteins as calculated by ImageJ. The relative average intensities ± standard deviation of pACLY/GAPDH normalized to siNC (F, *n* ≥ 2), E3/GAPDH (I, L, and M, *n* ≥ 3), D13/GAPDH (J, *n* ≥ 3), or A17/GAPDH (K, *n* ≥ 3), normalized to vehicle-treated, mock-infected cells, are shown in the tables underneath the images. n indicates the number of biological replicates.

Next, we examined the effects of inhibiting ACLY on VACV replication using SB 204990, a selective inhibitor of ACLY (58). Notably, SB 204990 treatment significantly reduced the VACV titers by 18- and 13-fold in cells infected at a multiplicity of infection (MOI) of 2 and 0.1, respectively (Fig. 4A), without affecting the cell viability (Fig. 4B).

To further corroborate these results, we treated the cells with two other chemical inhibitors of ACLY, BMS-303141 and NDI-091143 that inhibit ACLY with a different mechanism than that of SB 204990 (59) and assessed the virus replication. BMS-303241 and NDI-091143 reduced the VACV titers by 18- and 17-fold at a high MOI of 2 (Fig. 4C), and by 3,000- and 1,428-fold at a low MOI of infection (0.01) (Fig. 4D). The observed difference in reduction of VACV titers with three different inhibitors of ACLY could be due to different mechanisms of action of these compounds. While SB-204990 is a citrate-like inhibitor of ACLY (58), BMS-303241 and NDI-091143 are sulfonamides based (60). Importantly, none of the compounds affected the viability of HFFs at the concentration used in our experiments (Fig. 4E). Furthermore, we examined the genetic suppression of ACLY levels using small interfering RNAs (siRNAs). ACLY-specific siRNAs effectively reduced the protein expression levels of ACLY (Fig. 4F). ACLY silencing by two different siRNAs significantly suppressed VACV replication by 10-fold and 9-fold (Fig. 4G) without affecting cell viability (Fig. 4H). Taken together, these results demonstrated an important role of ACLY activity in VACV infection.

To determine the specific stage of viral replication affected, we measured the protein levels of VACV late (A17), intermediate (D13), and early (E3) proteins upon ACLY inhibition in HFFs with SB-204990 treatment. The treatment of VACV-infected HFFs with SB-204990 reduced the levels of VACV E3, D13, and A17 proteins (Fig. 4I through K) indicating that it affects VACV infection beginning at early time post-infection. Treatment of HFFs with BMS-303241 and NDI-091143 also reduced the levels of VACV early E3 protein levels (Fig. 4L).

To get a more precise picture of how ACLY inhibition is affecting VACV replication, we added SB-204990 at different times post-infection [0 (at the time of infection), 1, 2, and 3 hpi] and measured E3 protein levels at 4 hpi. We found that the addition of SB-204990 at the time of infection reduced the early protein levels more and the inhibitory effect was gradually reduced as the compound was added later after infection (Fig. 4M). This result indicates that ACLY inhibition likely starts to suppress VACV replication at a time point prior to early gene expression and likely multiple steps of VACV replication are affected.

Next, we tested whether the inhibition of ACLY had any effect on VACV entry into the cells. To test this, we used a qPCR-based assay to quantify the viral DNA that enter the cells at 1 hpi. Blebbistatin, which inhibits VACV entry (61) by inhibiting myosin-II-dependent blebbing, virus movement along filopodia, and macropinocytosis (62, 63), was used as a positive control. We found that the ACLY inhibitor, SB-204990 treatment, significantly reduced the VACV DNA in the cells at 1 hpi and that these levels were comparable to that of the entry inhibitor blebbistatin (Fig. 4N). These results indicate that ACLY inhibition can suppress VACV entry into the cells. The results also suggest that ACLY may have an additional role in VACV replication other than lipid metabolism at post-entry stages. Further experiments are required to confirm if VACV binding or fusion is also affected by ACLY inhibition.

### Growth factor signaling is needed for the VACV-mediated upregulation of ACLY phosphorylation

**Fig 5:**
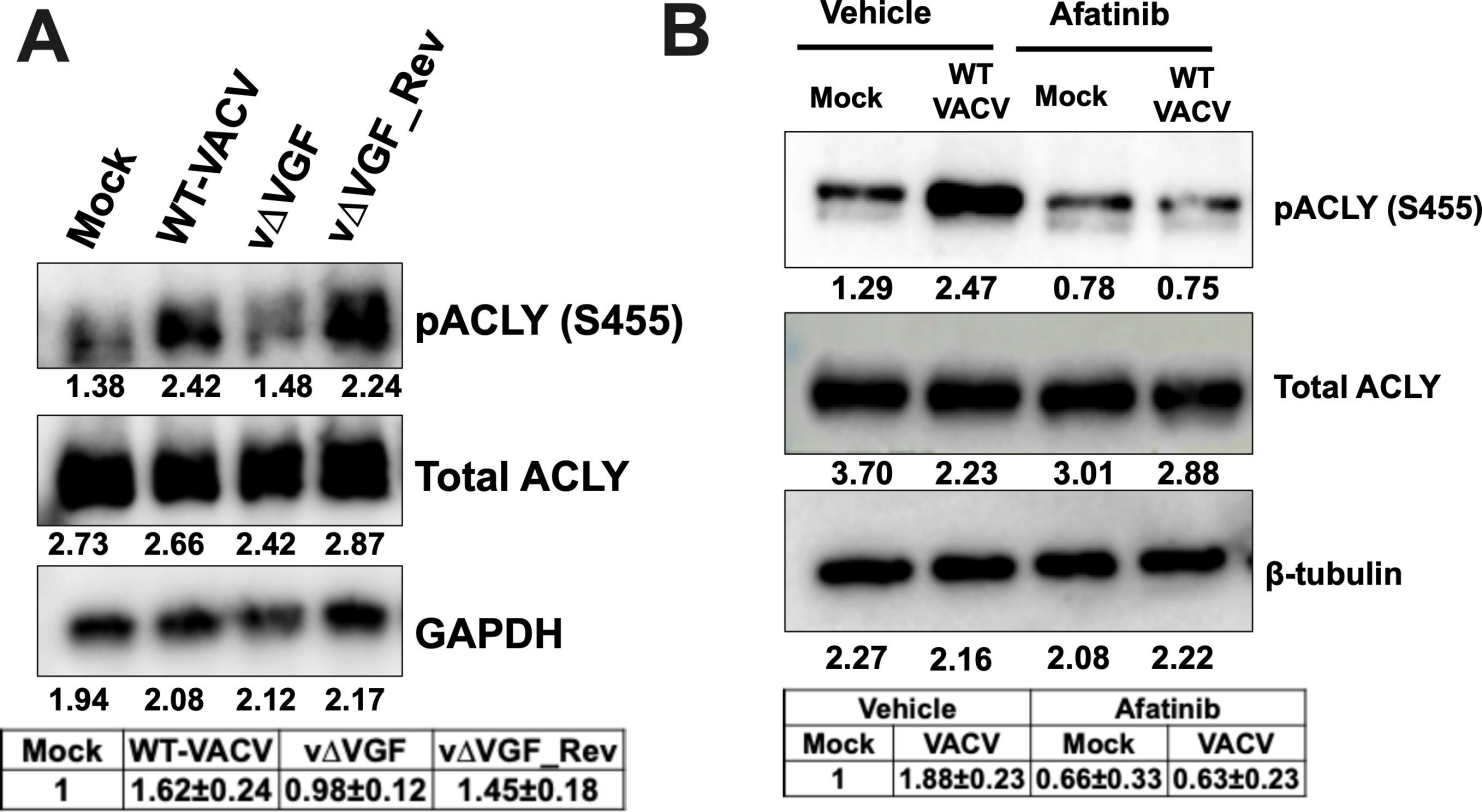
VACV infection induces ACLY S455 phosphorylation in a VGF-dependent manner. (**A**) VGF is crucial for the activation of ACLY phosphorylation (S455). HFFs were infected with the indicated viruses at an MOI of 5. Western blotting analysis was performed to measure the levels of ACLY at 4 h post-infection (hpi). GAPDH was used as a loading control. (**B**) VGF-induced epidermal growth factor receptor (EGFR) signaling is required to activate ACLY phosphorylation in VACV-infected cells. Uninfected or HFFs infected with VACV at an MOI of 5 in the presence or absence of 3 µM afatinib were used to detect ACLY levels by Western blotting at 4 hpi. β-tubulin was used as a loading control. Representative images of multiple biological replicates were shown. The numbers below each band indicate the average intensities of respective proteins as calculated by ImageJ. The relative average intensities ± standard deviation of pACLY/GAPDH or β-tubulin normalized to the mock vehicle are shown in the tables underneath the images (*n* ≥ 3). n indicates the number of biological replicates.

Due to the observed increase in ACLY phosphorylation early during VACV infection, we hypothesized that a viral early protein could be involved. VGF is one of the most highly expressed genes among the 118 VACV early genes (64, 65). Importantly, because we previously identified VGF as a key player in the upregulation of citrate levels in VACV-infected cells (10), we tested the role VGF plays in this process. We used a recombinant VACV from which both copies of the VGF gene were deleted (vΔVGF) from the inverted terminal repeats of the VACV DNA. We have previously shown that VACV early gene expression is not affected by the deletion of VGF (10). We also used a VGF revertant VACV (vΔVGF_Rev) by inserting one copy of the VGF gene under its natural promoter but at a different locus in the viral genome (10). Infection with vΔVGF abolished this upregulation of ACLY phosphorylation (Fig. 5A). Notably, phosphorylation could be rescued by infection with vΔVGF_Rev, indicating that VGF is required to induce ACLY phosphorylation (Fig. 5A).

Because VGF deletion renders VACV unable to increase ACLY phosphorylation, we surmised that VGF-mediated EGFR signaling is involved in the upregulation of ACLY phosphorylation. To explore this possibility, we first tested the effects of an irreversible EGFR inhibitor, afatinib (66), on ACLY levels at a concentration that was previously shown not to affect HFF viability (10). Afatinib treatment reduced the increase in ACLY phosphorylation in VACV-infected cells, with minimal effects observed on uninfected controls (Fig. 5B). Combined with the previous findings from our laboratory and others, showing significant reductions in VACV titers following the inhibition of the EGFR pathway (10, 67), our current results indicate that VGF-induced EGFR signaling-induced ACLY phosphorylation is required for efficient VACV replication.

### VACV infection upregulates ACLY phosphorylation in an Akt-signaling-dependent manner

**Fig 6:**
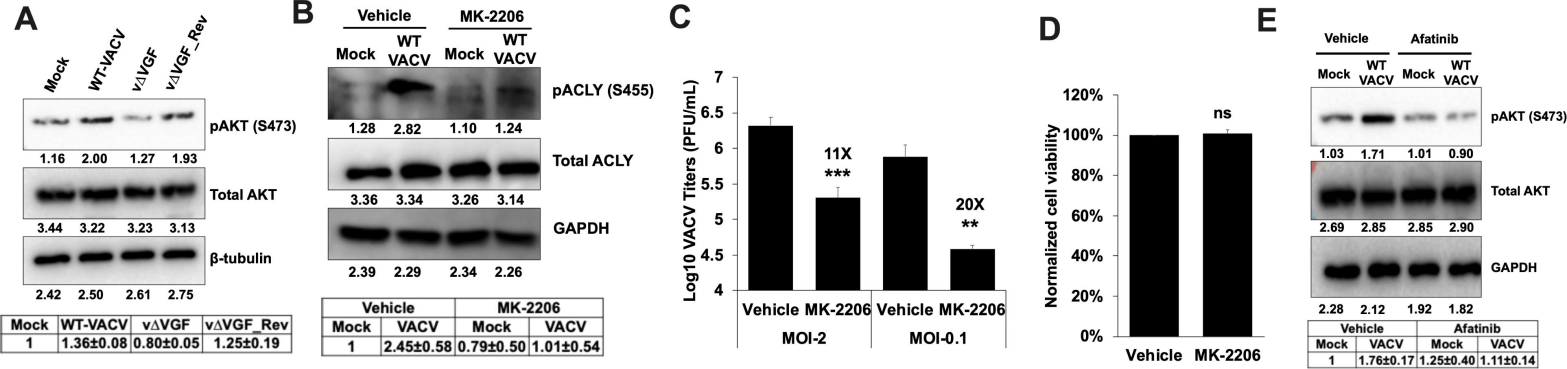
VACV infection upregulates ACLY phosphorylation in an Akt-dependent manner. (**A**) VACV infection induces protein kinase B (Akt) phosphorylation in a VGF-dependent manner. HFFs were infected with the indicated viruses at MOI of 5 for 2 h. Western blotting analysis was performed to measure the levels of phosphorylated or total Akt. (**B**) Akt inhibition suppresses ACLY phosphorylation under VACV-infected conditions. HFFs infected with MOI 5 of WT VACV (or uninfected controls) in the presence or absence of 5 µM MK 2206 (Akt inhibitor) were used to detect ACLY levels by Western blot at 4 hpi. (**C**) The inhibition of the Akt suppresses VACV replication. HFFs were infected with WT-VACV in the presence or absence of 5 µM MK 2206. Virus titers were measured by a plaque assay at 24 hpi (MOI = 2) and 48 hpi (MOI = 0.1). The numbers above the bars indicate the fold reduction in titers compared to vehicle (*n* ≥ 3). (**D**) The inhibition of Akt does not affect HFF viability. HFFs were cultured in the presence or absence of 5 µM MK 2206 for 48 h. Cell viability was determined by trypan blue exclusion assay (*n* ≥ 3). (**E**) The inhibition of EGFR signaling suppresses Akt phosphorylation upon VACV infection. Uninfected control or HFFs infected with WT VACV at an MOI of 5 in the presence or absence of 3 µM afatinib were used to measure Akt levels by western blotting assay at 4 hpi. Error bars represent the standard deviation of at least three biological replicates. For *P* values, ns, *P*  >  0.05; **P ≤ 0.01; ***P ≤ 0.001. For Western blot, representative images of multiple biological replicates were shown. The numbers below each band indicate the average intensities of respective proteins as calculated by ImageJ. The relative average intensities ± standard deviation of pAKT/β-tubulin (A, *n* ≥ 3), pACLY/GAPDH (B, *n* ≥ 3), and pAKT/GAPDH (E, *n* ≥ 3) normalized to the mock vehicle are shown in the tables underneath the images. n indicates the number of biological replicates.

Growth factors activate the PI3K-Akt cascade to elicit a variety of cellular functions (68). Akt is the predominant activator of ACLY phosphorylation (30, 57). Interestingly, VACV infection is known to activate Akt phosphorylation in mouse A31 cells and mouse embryonic fibroblasts, which could be observed at an early post-infection time point (69) and appears to be VGF-dependent in HFFs (70) (Fig. 6A).

We, therefore, examined whether Akt is necessary for the induction of ACLY phosphorylation in VACV-infected HFFs. We measured the levels of ACLY phosphorylation in uninfected and VACV-infected HFFs treated with MK-2206, a highly selective Akt inhibitor (71). MK-2206 treatment reduced ACLY phosphorylation in both uninfected and VACV-infected conditions (Fig. 6B). The reduction in the uninfected control was less pronounced than that observed in VACV-infected HFFs (Fig. 6B), highlighting the important role of Akt in ACLY phosphorylation during VACV infection.

To assess the effect of inhibition of Akt, which is a potent activator of various cellular functions including ACLY phosphorylation, on VACV replication we measured VACV titers upon chemical inhibition of Akt. The inhibition of Akt using MK-2206 significantly reduced VACV titers by 11- and 21-fold at MOI of 2 and 0.1, respectively (Fig. 6C), without affecting HFF viability (Fig. 6D). The findings agree with a previous report showing a reduction of VACV titers upon Akt inhibition in A31 cells and mouse embryonic fibroblasts (69). We further tested the effects of EGFR inhibition on Akt levels during VACV infection. The inhibition of EGFR suppressed Akt phosphorylation in VACV-infected cells but not in uninfected controls (Fig. 6E). Taken together, these results indicate that the VGF-induced EGFR pathway serves as an upstream activator of Akt phosphorylation during VACV infection, which results in increased ACLY phosphorylation.

### Growth factor signaling and ACLY are important for the VACV-mediated upregulation of neutral lipid droplet synthesis

**Fig 7:**
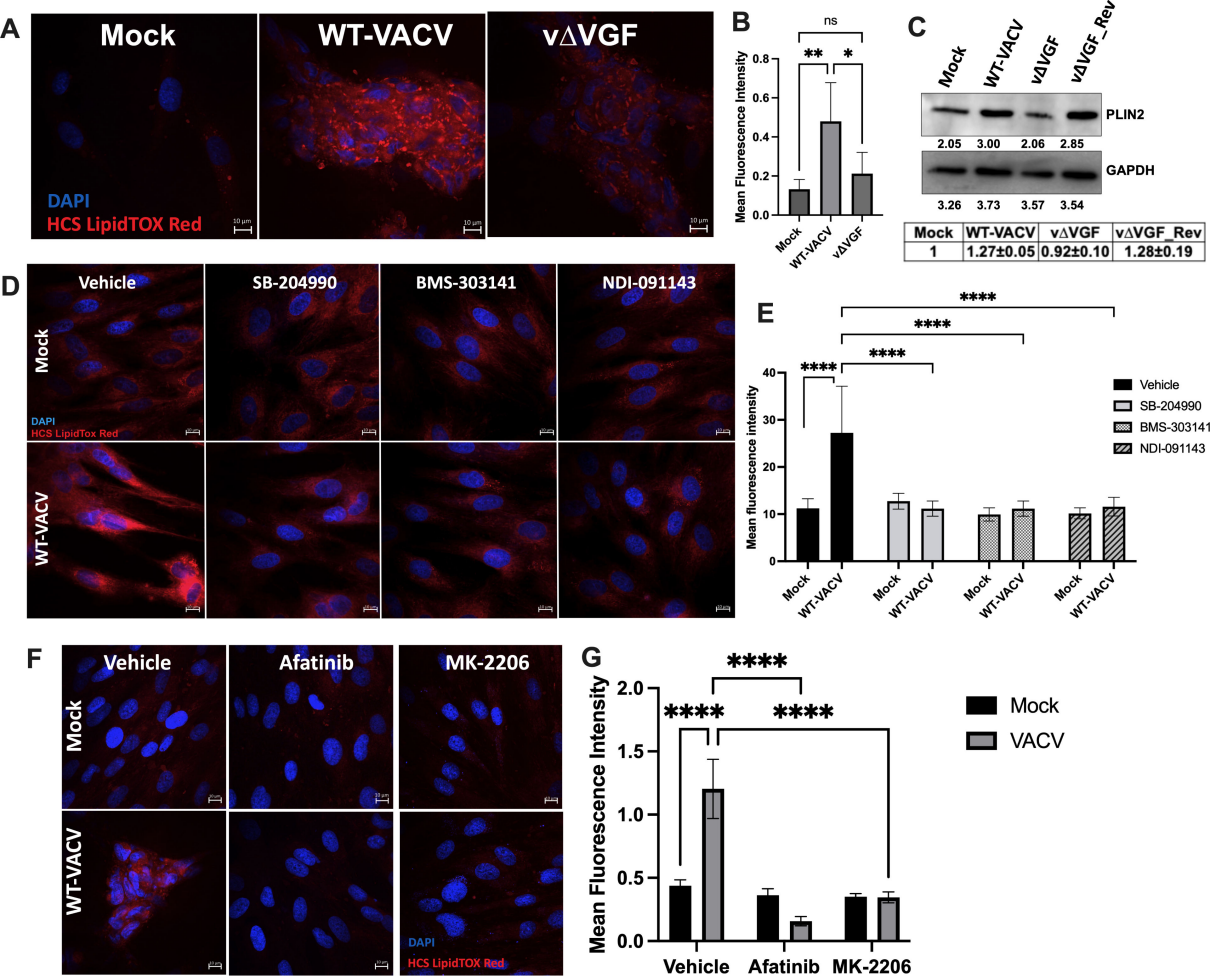
VGF-EGFR-Akt-ACLY signaling axis is required for stimulating neutral lipid synthesis during VACV infection. (**A**) VACV infection induces lipid droplet formation in HFFs in a VGF-dependent manner. HFFs were infected with the indicated viruses at MOI of 5 for 8 h. Lipid droplets were stained with HCS Lipidtox Red, and the nuclei were stained with DAPI and imaged under a confocal microscope (*n* ≥ 3). Although the images were taken at exact settings, the nuclear size could have appeared different because of the rounding of the VACV-infected cells. (**B**) The intensities of the red signals corresponding to the lipid droplets in (Fig. 7A) were quantified in the bar graph (*n* ≥ 3). (**C**) VACV infection increases the lipid droplet-associated protein levels in HFFs in a VGF-dependent manner. HFFs were infected with the indicated viruses at an MOI of 5 for 8 h. Western blotting analysis was performed to measure the levels of PLIN2. The numbers below each band indicate the average intensities of respective proteins as calculated by ImageJ. The relative average intensities ± standard deviation of PLIN2/GAPDH normalized to the mock vehicle are shown in the table underneath the images (*n* ≥ 3). (**D**) ACLY inhibition results in a decrease in VACV-induced lipid droplets. HFFs were infected with wildtype VACV at an MOI of 5 in the absence or presence of 100 µM SB-204990, 10 µM BMS-303141, or 20 µM NDI-091143 for 8 h. Lipid droplets were stained with HCS Lipidtox Red, and the nuclei were stained with DAPI and imaged under a confocal microscope (*n* ≥ 3). (**E**) The intensities of the red signals corresponding to the lipid droplets in (Fig. 7D) were quantified in the bar graph (*n* ≥ 3). (**F**) EGFR and Akt pathways are important for the formation of lipid droplets during VACV infection. HFFs were infected with wild-type VACV at a MOI of 5 for 8 h in the presence or the absence of 3 µM afatinib (EGFR inhibitor) or 5 µM MK-2206 (Akt inhibitor). Lipid droplets were stained with HCS Lipidtox Red and imaged under a confocal microscope. (**G**) The intensities of the red signals corresponding to the lipid droplets in (Fig. 7F) were quantified in the bar graph (*n* ≥ 3). Error bars represent the standard deviation of at least three biological replicates. For *P* values, *P ≤ 0.05; **P ≤ 0.01; ****, *P* ≤ 0.0001. n indicates the number of biological replicates.

Next, we investigated the role of the VACV VGF-EGFR-Akt-ACLY signaling axis in inducing lipid droplets upon infection. Our HCS Lipidtox staining showed that vΔVGF infection resulted in a lower level of lipid droplets compared to WT-VACV (Fig. 7A and B), indicating a vital role of VGF in neutral lipid synthesis. In line with these findings, the increase in PLIN2 levels infected with WT-VACV was not observed in cells infected with vΔVGF (Fig. 7C). Remarkably, PLIN2 levels were rescued by infection with vΔVGF_Rev (Fig. 7C), indicating that VGF is a crucial viral protein required to form lipid droplets. The chemical inhibitors of ACLY also significantly reduced the neutral lipid droplet formation that was induced upon VACV infection (Fig. 7D and E). Moreover, inhibition of the EGFR and PI3K-Akt pathway using specific inhibitors reduced the neutral lipid droplet levels upon VACV infection (Fig. 7F and G). Although the inhibitors used are highly selective, it is possible that inhibition of an upstream molecule like EGFR or Akt could have pleiotropic effects other than the intended cascades because of the inherent crosstalk in the signaling pathways. Nevertheless, our results show that the VGF-EGFR-Akt-ACLY pathway is required for stimulating neutral lipid synthesis in VACV infection.

### VGF and EGF peptides differentially regulate ACLY phosphorylation and PLIN2

**Fig 8:**
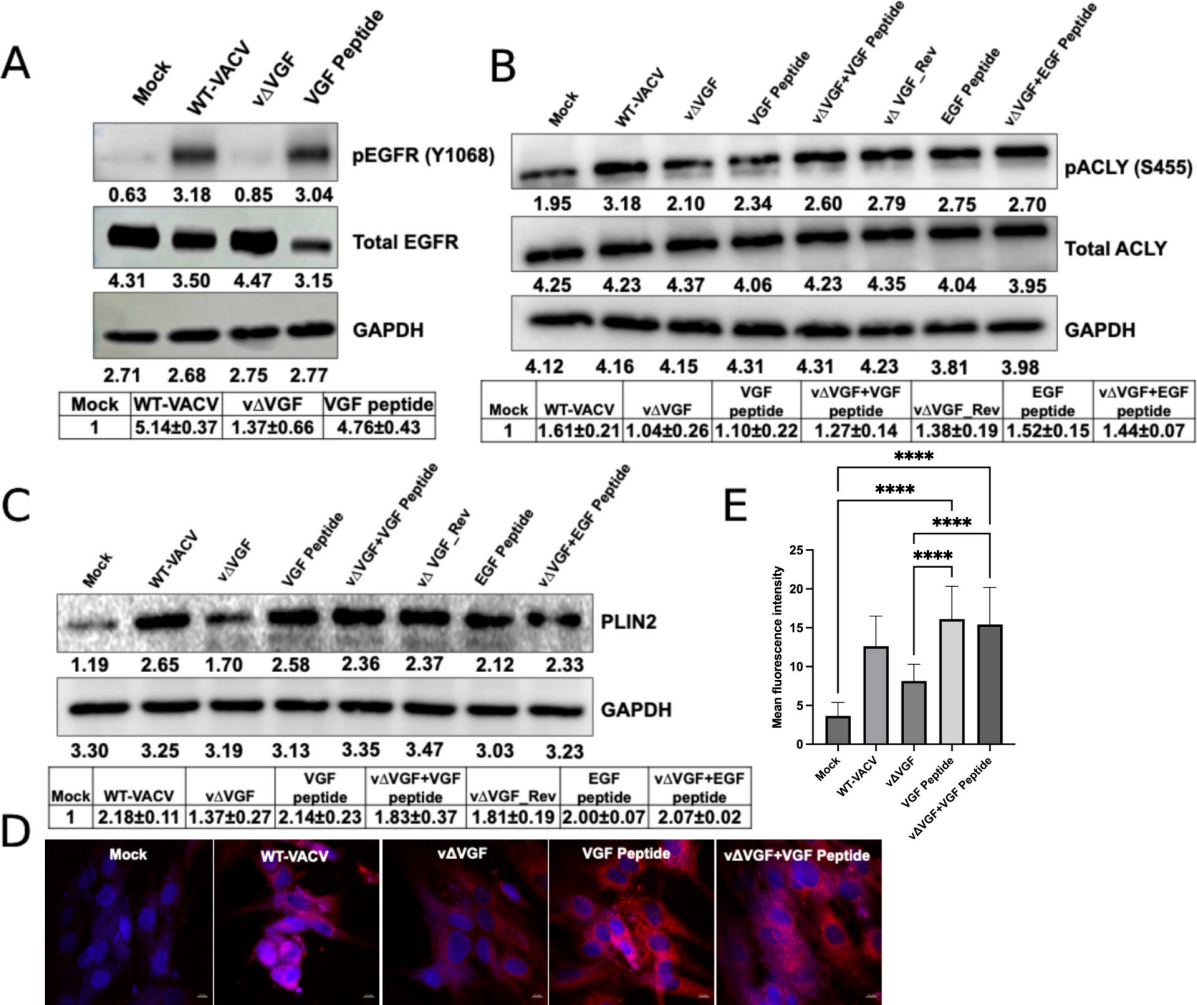
VGF and EGF peptides differentially regulate ACLY phosphorylation and neutral lipid synthesis in HFFs. (**A**) The treatment of purified, recombinant VGF peptide induces phosphorylation of EGFR in HFFs. HFFs were infected with the indicated viruses at an MOI of 2. Wherever indicated, the cells were treated with 5 µg/mL of VGF peptide. Western blotting analysis was performed to measure the levels of indicated proteins at 8 hpi. GAPDH was used as a loading control. (**B**) VGF peptide alone is not sufficient to induce ACLY phosphorylation in HFFs. HFFs were infected with the indicated viruses at an MOI of 2. Wherever indicated, the cells were treated with 5 µg/mL of VGF or EGF peptide with or without infection. Western blotting analysis was performed to measure the levels of indicated proteins at 8 hpi. GAPDH was used as a loading control. (**C**) VGF peptide alone can induce PLIN2 in HFFs. HFFs were infected with the indicated viruses at an MOI of 2. Wherever indicated, the cells were treated with 5 µg/mL of VGF or EGF peptide with or without infection. Western blotting analysis was performed to measure the levels of indicated proteins at 8 hpi. GAPDH was used as a loading control. (**D**) VGF peptide treatment induces the levels of neutral lipid droplets. HFFs were infected with the indicated viruses at an MOI of 2. Wherever indicated, the cells were treated with 5 µg/mL of VGF peptide with or without infection. Lipid droplets were stained with HCS Lipidtox Red and imaged under a confocal microscope at 16 hpi. (**E**) The intensities of the red signals corresponding to the lipid droplets in Fig. 8D were quantified in the bar graph (*n* ≥ 3). Error bars represent the standard deviation of at least three biological replicates. For *P* values, *P ≤ 0.05; **P ≤ 0.01; ****P ≤ 0.0001. For Western blotting analyses, representative images of multiple biological replicates were shown. The numbers below each band indicate the average intensities of respective proteins as calculated by ImageJ. The relative average intensities ± standard deviation of pEGFR/GAPDH (A, *n* ≥ 3), pACLY/GAPDH (B, *n* ≥ 3), PLIN2/GAPDH (C, *n* ≥ 2) normalized to mock are shown in the tables below the images. n indicates the number of biological replicates.

Finally, we aimed to test whether the VGF and EGF peptides alone can support the phosphorylation of ACLY and PLIN2. To this end, we treated the cells with a peptide corresponding to the sequence of the cleaved, secreted portion of the VGF (expressed and purified from mammalian cells) peptide, which stimulated EGFR similar to WT-VACV infection (Fig. 8A), indicating its biological activity. Interestingly, VGF peptide alone was not sufficient to induce ACLY phosphorylation in uninfected HFFs (Fig. 8B). ACLY phosphorylation in VGF peptide plus vΔVGF-infected conditions was higher than that of uninfected or vΔVGF-infected cells (Fig. 8B). These results indicate that although VGF is important for ACLY phosphorylation during VACV infection, the VGF peptide alone is insufficient and needs additional factor(s) to fully induce ACLY S455 phosphorylation. On the other hand, EGF peptide alone was able to induce ACLY phosphorylation (Fig. 8B). However, the treatment with either VGF or EGF peptide alone was able to increase the levels of the lipid droplet-associated protein PLIN2 (Fig. 8C)**,** further corroborating the important role of EGFR pathway in lipid droplet synthesis. In addition, the treatment of VGF peptide alone was able to increase the levels of HCS Lipidtox staining in HFFs (Fig. 8D and E), indicating the sufficiency of VGF peptide in inducing neutral lipid droplets. These findings suggest that, in addition to ACLY, other factor(s) may be involved in promoting neutral lipid droplet formation in HFFs. Taken together, while both VGF and EGF can participate in the same pathways, their efficiencies and possibly mechanisms of action vary, leading to different effects on ACLY phosphorylation and PLIN2 expression.

## DISCUSSION

**Fig 9:**
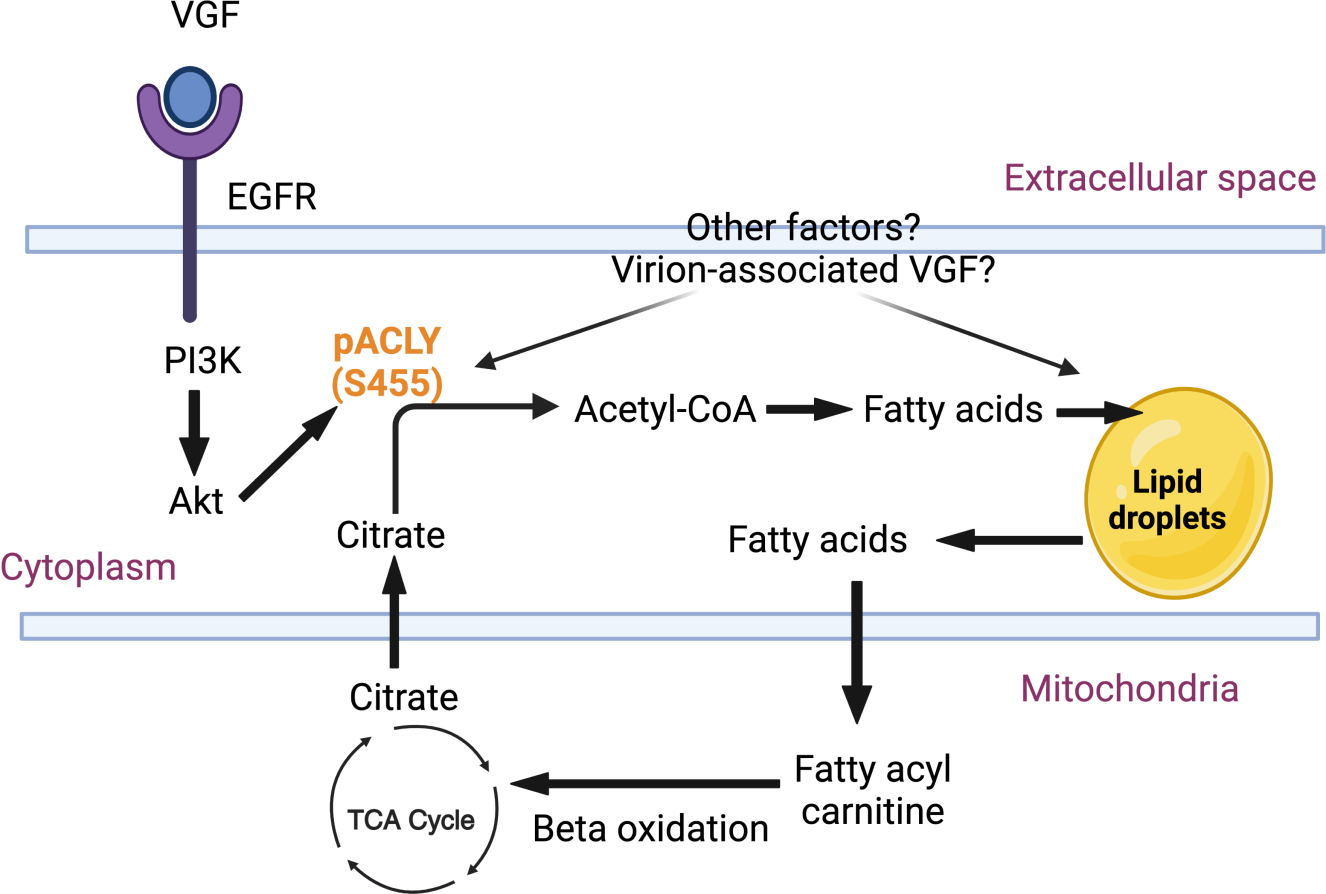
Proposed mechanism and biological impact of induction of lipid droplets during the infection of HFFs with VACV. VACV infection increases ACLY phosphorylation in a VGF-EGFR-PI3K/Akt-dependent fashion that leads to increased neutral lipid droplet formation, geared toward generating β-oxidation intermediates that are eventually recycled to the TCA cycle to generate energy. Other viral and cellular factors may be involved in the induction of ACLY phosphorylation and neutral lipid metabolism, warranting further study for full characterization. Created with BioRender.com.

In the present study, we discovered that ACLY, previously unrecognized as a target of viral modulation, is stimulated by VACV infection. This infection enhances ACLY S455 phosphorylation and lipid droplet formation, with evidence suggesting dependency on VGF, the VACV homolog of cellular EGF. EGFR-induced Akt phosphorylation is crucial for increasing ACLY phosphorylation during VACV infection. In addition, VGF-EGFR-Akt-ACLY signaling axis is essential for neutral lipid droplet formation, which could be converted to fatty acylcarnitine to feed the TCA cycle for bioenergetic requirements (Fig. 9). Together with our previous finding that VGF-induced EGFR activates non-canonical STAT3 phosphorylation to increase TCA cycle intermediate levels (10), these findings highlight VGF’s multiple roles in rewiring host metabolism during VACV infection by modulating various cellular signaling pathways and metabolic processes.

VACV induces profound alterations of metabolism in its host cells including oxidative phosphorylation (OXPHOS), the oxygen consumption rate (OCR), and the TCA cycle metabolites (10, 12, 72, 73). Here, we unveiled the molecular mechanisms underlying the VACV-mediated modulation of a key step linking the TCA cycle and neutral lipid metabolism. VACV infection increases ACLY phosphorylation in a VGF-dependent manner. ACLY sits at the crossroads of the TCA cycle, fatty acid metabolism, and glutamine metabolism (Fig. 1A). Interestingly, VACV induces changes in all three aspects of cell metabolism, suggesting that ACLY is a key regulator in the mediation of VACV-host interactions at the metabolic interface. We have previously shown that VACV infection increases the levels of TCA cycle intermediates (10). Paradoxically, despite the increased phosphorylation of the catalyst (ACLY), VACV infection appears to induce the production of higher levels of the reactant (citrate) and lower levels of the product (Acetyl-CoA) (10). Upon VACV infection, we did not observe a significant increase in the protein levels of the mitochondrial citrate transporter (SLC25A1; not shown) or any obvious increases in levels of ACC1, which catalyzes the irreversible carboxylation of Acetyl-CoA required for fatty synthesis (Fig. 3B). Furthermore, an overall decrease in the steady-state levels of long-chain fatty acids was observed following VACV infection (10). Long-chain fatty acids are acylated and then carnitylated by carnitine palmitoyltransferase (CPT1) and are then transported into the mitochondrial matrix, where they undergo β-oxidation to fuel the TCA cycle (74). We have previously shown that the levels of carnitylated fatty acids increase following VACV infection, and inhibition of fatty acid β-oxidation suppresses VACV replication indicating an important role of VACV-induced upregulation of fatty acid β-oxidation in a VGF-dependent manner (10). It is known that ACLY positively regulates the carnitine system (75). Our findings suggest that the observed increase in ACLY phosphorylation in VACV-infected cells (Fig. 3C and D) is necessary for increased neutral lipid droplets formation (Fig. 1B through D and 7A through C), geared toward generating β-oxidation intermediates (10) that could be eventually recycled to the TCA cycle to generate energy (10–12).

Our results raise an intriguing question: why does VACV go to such lengths to upregulate the levels of TCA cycle intermediates and the neutral lipid droplets? By upregulating ACLY phosphorylation and redirecting the host metabolism toward neutral lipid droplet formation, VACV could achieve multiple goals. First, because VACV is an enveloped virus, it requires lipid molecules to synthesize its membrane (76). The lipids derived from the envelope of neutral lipid droplets may be used during virion morphogenesis. Second, the fatty acids derived from the neutral lipid droplets could provide the essential intermediates to generate β-oxidation substrates for maintaining an energy-rich state to support the increased demands associated with viral replication (12). Third, as seen in other viruses (16), lipid droplets could play vital roles in facilitating the magnitude of the early antiviral immune response during VACV infection. The formation of lipid droplets is important for efficient VACV replication (Fig. 2A, B, and F). Because lipid droplets are highly dynamic organelles, further studies are warranted to decipher the exact functions of VACV-induced lipid droplets in virus replication.

While ACLY and neutral lipid droplets are important for VACV replication, interestingly, ACLY plays an additional role starting early during the VACV life cycle including viral entry (Fig. 4I through K, M, and N). ACLY is important for macropinocytosis (77), a non-selective form of endocytosis, which is one of the mechanisms VACV uses for entry (63). Further studies are needed to examine how ACLY affects VACV entry into the cells, in addition to the roles at the post-entry steps. While lipid droplet formation is also dependent on ACLY (Fig. 7G and E), its inhibition affects only the later stages of VACV replication (Fig. 2F). These results suggest that lipid droplet synthesis is one of the effects of metabolic reprogramming during VACV infection. It is possible that the VACV-mediated activation of ACLY could affect fatty acid metabolism beyond lipid droplets such as phospholipids and cholesterol. The chemical or genetic inhibition of ACLY, thus, could have broader impacts on the replication of VACV as compared to the inhibitors of lipid droplet synthesis alone. Further studies are needed to identify if other metabolic processes are dependent on the VACV-ACLY pathway and are affected on ACLY inhibition.

VGF is important for the phosphorylation of ACLY and induction of neutral lipid droplet synthesis during VACV infection. However, the VGF peptide alone was not sufficient to induce ACLY phosphorylation in uninfected HFFs. This insufficiency potentially stems from its dependence on viral infection context, requiring additional viral factors, cooperative signaling pathways, an altered cellular state, or some unknown autocrine/paracrine effects that are present during virus infection. The differential effects of the VGF peptide on PLIN2, neutral lipid droplets, and ACLY phosphorylation likely reflect the involvement of distinct mechanisms or signaling pathways, with PLIN2 regulation being more directly influenced by VGF, while ACLY phosphorylation requires a more complex set of conditions or interactions. It is also possible that VGF may have evolved to lose the ability to stimulate ACLY in the absence of infection as stimulation of uninfected cells may promote cell proliferation, which may, in turn, compete with VACV for nutrition, especially when the nutrition is limited. EGF peptide has a stronger inherent ability to induce the necessary cooperative signaling pathways independently, which would explain its higher effectiveness in activating ACLY phosphorylation.

We found that ACLY phosphorylation is markedly reduced in VACV-infected cells upon inhibition of protein synthesis with CHX treatment but not upon DNA synthesis with AraC (not shown), indicating early viral protein expression is required for the upregulation of ACLY activities during VACV infection. This also provides a possible explanation as to why the synthesized secreted VGF alone did not show a significant effect on ACLY phosphorylation. VGF has been detected in purified VACV virions (78), though it is less likely a virion component. It is possible that a small amount of VGF can be co-purified with virions due to its high expression. Despite this, VGF in the virion most likely does not interfere with signaling from newly synthesized VGF, as VACV infection activates ACLY phosphorylation from the early stages of infection, persisting until at least 8 h post-infection.

While lipid droplet formation has been observed primarily in macrophage models following bacterial infection, only a limited number of studies have been carried out in viral infections. Some positive-stranded RNA viruses, such as sindbis and dengue viruses, induce lipid droplet formation in mosquito midgut cells (79). In another study, infection of mammalian cells with herpes simplex virus-1, influenza A virus, dengue virus, and Zika virus showed transient induction of lipid droplets early during virus replication that corresponded with the detection of intracellular dsRNA and dsDNA (16). Interestingly, this induction of lipid droplets is independent of type-I interferon (IFN) and dependent on the EGFR-PI3K pathway. Because VACV infection produces dsRNA during the transcription of viral intermediate or late genes (80), it would be interesting to examine whether the VGF-induced EGFR-PI3K pathway and dsRNA work in conjunction to enhance lipid droplet formation during VACV replication. Furthermore, a recent report indicates that ISG15, an interferon-stimulated gene, is required for inducing lipid droplet formation during VACV infection of mouse bone marrow-derived macrophages (BMDM) (21). It is important to note that VACV encodes dozens of immune regulators and efficiently blocks interferon responses during productive infection of many cell types including HFFs (81–83). It remains to be tested whether ISG15 is also involved in the VGF-EGFR-ACLY signaling cascade to induce neutral lipid biosynthesis, and if so, whether it functions upstream or downstream of ACLY.

ACLY is not the sole source of acetyl-CoA and is not exclusively localized to the cytosol (84). During nutrient-restricted conditions, such as starvation, the enzyme ACCS2 can convert acetate into acetyl-CoA (85, 86). During human cytomegalovirus (HCMV) infection, the loss of the ability to utilize citrate for Acetyl-CoA synthesis through ACLY has little effect on either lipid synthesis or viral growth because ACCS2 compensates for the loss of ACLY (87). Because acetate supplementation did not enhance VACV replication (not shown) and ACLY inhibition severely suppressed viral replication, the function of ACCS2 appears unlikely to be of similar importance as ACLY during VACV infection. Although ACLY is a predominantly cytosolic enzyme, several studies have reported its localization to the nucleus (84, 88). The acetyl-CoA generated in the nucleus by nuclear ACLY is vital for homologous recombination (88) and histone acetylation (84). Further studies remain necessary to determine the intracellular distribution of ACLY during VACV infection and the effects, if any, of altered localization patterns on the modulation of transcription.

Our current understanding of the role of ACLY in virus replication is limited. While chemical and genetic inhibition of ACLY suppressed the replication of SARS-CoV-2 (89), indicating it could be an important host factor governing the replication of the virus, it is yet unclear if SARS-CoV-2 increases the phosphorylation or activity of ACLY. Another study in transformed hepatocellular carcinoma cells and transgenic mice expressing the Hepatitis B virus (HBV) pre-S2 mutant in the liver showed increased ACLY phosphorylation through mTOR signaling to induce the levels of neutral lipids such as triglycerides and cholesterol (90), without showing a direct effect of HBV infection on ACLY levels. In this regard, our finding sets a precedent to explore the effect of other viruses in modulating this critical host enzyme and how it affects their metabolism and replication. This will also open new avenues to develop novel targets for antiviral therapy.

In conclusion, this study identified the VGF-EGFR-Akt-ACLY pathway as a required axis for the synthesis of lipid droplets that is crucial for VACV replication. Because poxviruses are widely used to develop oncolytic agents (4), and the ACLY-induced metabolism is often dysregulated in cancer cells (26, 35), our findings could lead to improvement in poxvirus-based oncolytic virotherapy and the development of better antipoxvirus agents.

### Limitations of the study

It has been suggested that S455 phosphorylation contributes to the activation of ACLY enzyme activity (56, 57, 91–93). However, this could be context dependent, and cell-type specific. It has been established for bacterially expressed protein and may not be a rate-limiting factor during other events such as allosteric regulation of ACLY by sugars (28). It would be interesting to investigate VACV replication and its effect on metabolism in the S455 mutated cells to establish a direct role of this phosphorylation during VACV infection. It also remains to be tested whether VACV infection alters ACLY phosphorylation at other sites.

While we and others have shown that the protein levels of the enzymes ACAT, DGAT1, FASN, and ACC are not affected during VACV infection, it does not rule out the possibility that their activities are modulated in infected versus uninfected HFFs. A study of the enzyme kinetics of the proteins involved in the neutral lipid synthesis pathway could provide an informative picture.

## Data Availability

The data include Western blotting analyses, virus titration, PCR, luciferase luminescence activities, and microscopic fluorescent images. The authors confirm that the data supporting the findings of this study are available within the article and the raw data are available upon request. There are no large data sets included in this paper.
